# Entanglements of structure elements revealed in RNA 3D models

**DOI:** 10.1093/nar/gkab716

**Published:** 2021-08-25

**Authors:** Mariusz Popenda, Tomasz Zok, Joanna Sarzynska, Agnieszka Korpeta, Ryszard W Adamiak, Maciej Antczak, Marta Szachniuk

**Affiliations:** Institute of Bioorganic Chemistry, Polish Academy of Sciences, Noskowskiego 12/14, 61-704 Poznan, Poland; Institute of Computing Science & European Centre for Bioinformatics and Genomics, Poznan University of Technology, Piotrowo 2, 60-965 Poznan, Poland; Institute of Bioorganic Chemistry, Polish Academy of Sciences, Noskowskiego 12/14, 61-704 Poznan, Poland; Institute of Computing Science & European Centre for Bioinformatics and Genomics, Poznan University of Technology, Piotrowo 2, 60-965 Poznan, Poland; Institute of Bioorganic Chemistry, Polish Academy of Sciences, Noskowskiego 12/14, 61-704 Poznan, Poland; Institute of Computing Science & European Centre for Bioinformatics and Genomics, Poznan University of Technology, Piotrowo 2, 60-965 Poznan, Poland; Institute of Bioorganic Chemistry, Polish Academy of Sciences, Noskowskiego 12/14, 61-704 Poznan, Poland; Institute of Computing Science & European Centre for Bioinformatics and Genomics, Poznan University of Technology, Piotrowo 2, 60-965 Poznan, Poland; Institute of Bioorganic Chemistry, Polish Academy of Sciences, Noskowskiego 12/14, 61-704 Poznan, Poland; Institute of Computing Science & European Centre for Bioinformatics and Genomics, Poznan University of Technology, Piotrowo 2, 60-965 Poznan, Poland

## Abstract

Computational methods to predict RNA 3D structure have more and more practical applications in molecular biology and medicine. Therefore, it is crucial to intensify efforts to improve the accuracy and quality of predicted three-dimensional structures. A significant role in this is played by the RNA-Puzzles initiative that collects, evaluates, and shares RNAs built computationally within currently nearly 30 challenges. RNA-Puzzles datasets, subjected to multi-criteria analysis, allow revealing the strengths and weaknesses of computer prediction methods. Here, we study the issue of entangled RNA fragments in the predicted RNA 3D structure models. By entanglement, we mean an arrangement of two structural elements such that one of them passes through the other. We propose the classification of entanglements driven by their topology and components. It distinguishes two general classes, interlaces and lassos, and subclasses characterized by element types—loops, dinucleotide steps, open single-stranded fragments—and puncture multiplicity. Our computational pipeline for entanglement detection, applied for 1,017 non-redundant models from RNA-Puzzles, has shown the frequency of different entanglements and allowed identifying 138 structures with intersected assemblies.

## INTRODUCTION

Ribonucleic acids play vital roles in all living systems. They also constitute the genomes of most infectious viruses such as HIV, HCV, Ebola, influenza or SARS-CoV-2, fuelling recent worldwide pandemic. The discovery of numerous biological functions of these complex molecules is accompanied by the research into understanding their three-dimensional structure (1–3). And the knowledge of structure comes from experimental techniques—e.g. X-ray crystallography, NMR spectroscopy, cryo-microscopy, chemical probing (4–7)—and vividly developing computer prediction methods (8,9).

*In silico* modeling of RNA 3D structure has substantially developed in the recent decade (9). The semi- and fully automated predictions are complementary methods to the experimental determination of RNA folds. We could have observed their progress with the following challenges of RNA-Puzzles, the collective experiment to evaluate sequence-based RNA 3D structure prediction methods (8,10–13), and the follower of CASP (14). Careful assessment of numerous RNA 3D models submitted in nearly thirty Puzzles reveals the pros and cons of automated predictors and highlights the importance of integrating experimental data in the modeling process (12,15). Computer-generated structures often deviate from the native conformation both in terms of global folding and the formation of local regions. They are also often characterized by incorrect stereochemical parameters (16). Identification of these deviations and their origin allows prospecting how to remove major bottlenecks of RNA 3D structure prediction.

This work focuses on unusual fragment assemblies that RNA molecules do not appear to adopt while folding in their natural environment. We report on previously unpublished observations of structure elements’ entanglements in the predicted RNA 3D models. This type of entanglement occurs when a loop or a dinucleotide step is crossed by a sugar-phosphate backbone or paired bases that belong to another structural element. The problem was first perceived in a few prototypes generated by the RNAComposer system (17,18). In most cases, the method has rejected such entangled models because of their unfavorable energy, but some have slipped into the output sets. Further, similar entanglements have been found in RNA models built by the other algorithms (11,12). It has confirmed that the problem is general and not in the domain of just one algorithm for RNA structure prediction. Finally, a closer look at the experimental structures has revealed that some types of entanglements may occasionally occur in these structures as well (19), although these are rare cases.

In the paper, we describe the problem of entanglements, which can form between fundamental 3D structure elements of RNA molecules. We discuss potential types of entanglements and propose a new nomenclature for their unambiguous symbolic encoding. We sketch a workflow for the automatic detection of such conformations in RNA 3D structures, and we apply it to analyze the dataset of 1,050 models available within RNA-Puzzles resources (13). Finally, we show the distribution of different entanglements across the models and the frequency of their occurrence. These results will contribute to improving methods for RNA 3D structure prediction and allow building RNA models free from erroneous entanglements.

## DESCRIPTION OF THE PROBLEM

Entanglements we are concerned with occur in structured RNAs. We define them as spatial arrangements involving two structural elements, where at least one punctures the other. Puncture takes place when a structural element intersects the area within the other (closed) element. RNA structure elements are determined by the secondary structure of the molecule. Typically they include loops, stems, and single-stranded fragments. In our entanglement identification protocol, long double-stranded fragments are not treated holistically—each pair of the neighboring base pairs (i.e. dinucleotide step) defines a separate structure element. Loops and dinucleotide steps are referred to as closed structure elements, while single-stranded fragments are called open.

The tight dependence on the secondary structure is what differs the problem studied here from the problem of entangled protein and chemical polymer chains - the latter one has no coincidence to base pairing (20–22). Algorithms that identify knots in molecular structures examine the course of a polymer chain in the 3D space disregarding hydrogen bonds between the residues (23,24).

### Putative types of entanglements

Based on the entanglement’s topology, we distinguish two general classes: interlaces and lassos. The first type occurs when two closed structural elements form the Hopf link topology (25). Each involved element is then both punctured and the puncturing one. In the lasso-type arrangement, only one element is punctured. It embraces the other—the puncturing one.

**Figure 1.**
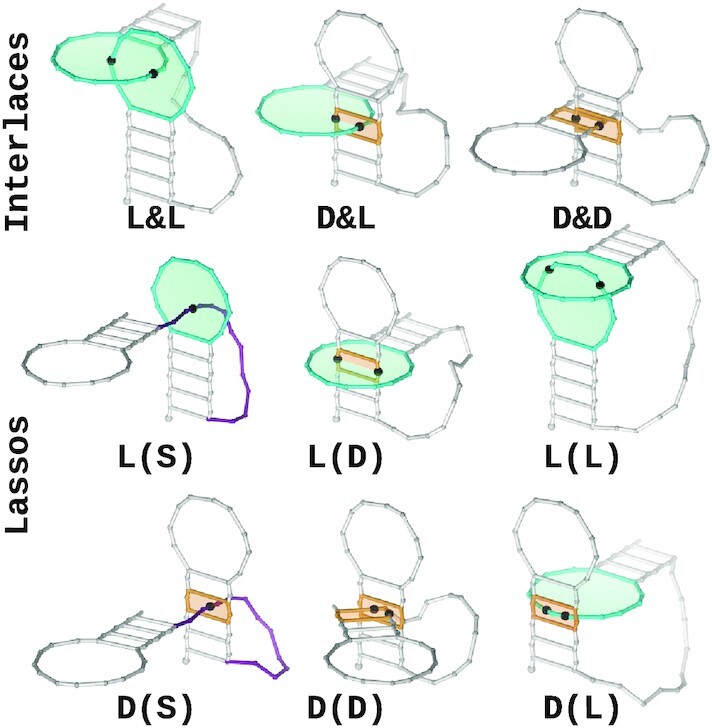
Putative types of RNA 3D structure entanglements are shown using simplified hairpin representations as an example. Entangled structure elements are color-coded: loops in cyan, dinucleotide steps in orange, and single-stranded fragments in magenta. Intersection sites are marked with black beads.

In both classes, we distinguish subclasses defined by the types of entangled elements—each is assigned a label, which consists of structure element symbols (L—loop, D—dinucleotide step, S—single-stranded fragment) and the parent class symbol (&—interlace, ()—lasso). Thus, for example, D&L denotes a dinucleotide step interlaced with a loop, while L(D) represents a dinucleotide step lassoed by a loop. Figure 1 displays schematic drawings of all types of structure elements’ entanglements.

## MATERIALS AND METHODS

### Test data set

The test set has been created from the RNA-Puzzles benchmark data (13). Initially, it has contained 1,050 RNA 3D structures from 22 challenges, including 22 targets determined by X-ray crystallography and 1,028 predictions (8,10–12). The data files have been download in January 2021 from the standardized dataset available at https://github.com/RNA-Puzzles and divided by challenges. The 233 of these RNA 3D models were predicted by web servers in the fully-automated processes (web server category). The remaining 795 structures were modeled in a hybrid approach, integrating automatic and manual methods (human category). In this dataset, we have found 33 redundant predictions and removed them, remaining with a collection of 1,017 RNAs for further analysis.

### Identification of entanglements

**Figure 2.**
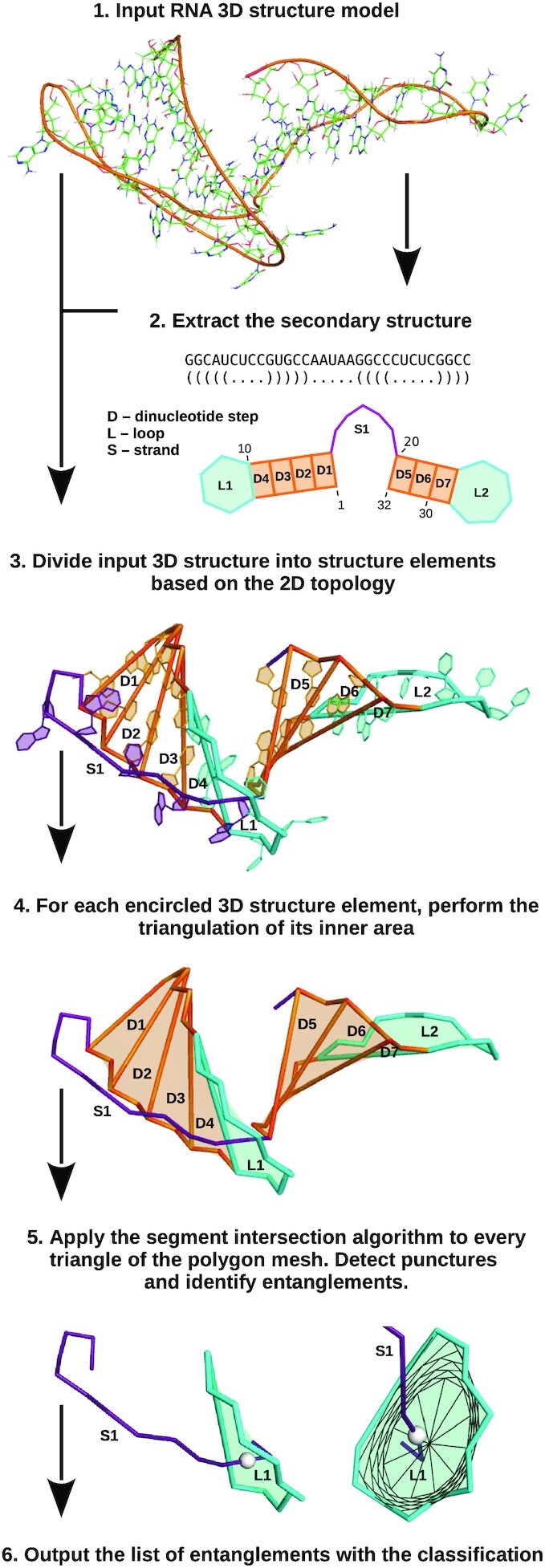
The pipeline for entanglement identification in RNA 3D structures.

We have developed an original computational pipeline to identify entanglements in the RNA 3D structures. It involves the sequential running of several algorithms, some of them derived from our other tools (Figure 2). First, we extract the secondary structure of the input RNA molecule, considering pseudoknots of different orders (26,27). In this step, we use procedures implemented in the RNApdbee system (28–30). Based on the secondary structure, we partition the input 3D model into open and closed structural elements. Here, we apply an extended version of the fragmentation algorithm that was designed for RNA FRABASE and RNAComposer systems (31–33). By the open structural element, we understand a coherent fragment of RNA strand that is not closed by canonical base pair - for example, dangling 5′- or 3′-end. The closed element of an RNA 3D structure is a circular conformation created either by a single-stranded fragment closed by canonical base pair or *n* single-stranded fragments linked by *n* canonical base pairs. Closed structural elements include loops (hairpin loops, multi-loops, internal loops, bulge loops) and dinucleotide steps. In some applications, the latter ones are also classified as loops (named stacking loops)—e.g. in the energy models used by the RNA secondary structure prediction programs (34,35). For every structure element, we create a polygonal chain that passes through selected atoms of the backbone (P, C4’) and the centroids of heavy atoms forming Watson–Crick hydrogen bonds. In closed elements, it encircles an area that lies within. This area is covered with a polygon mesh via recursive triangulation procedure. For every triangle, we apply the Moeller algorithm (36,37) to detect segment intersection sites, hereafter referred to as punctures. For each puncture, we identify two structural elements - the puncturing and the punctured one. It allows us to determine the type of entanglement. Finally, we create a list of entanglements.

## RESULTS AND DISCUSSION

### Statistics for the test set

**Figure 3.**
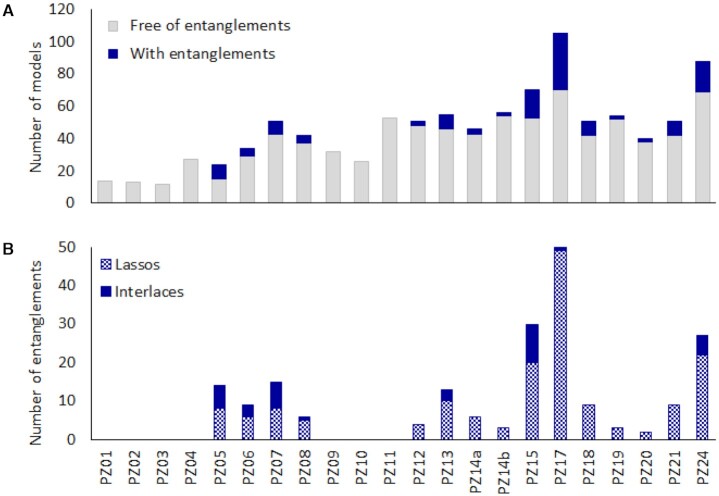
The number of (**A**) non-redundant models with and without entanglements predicted within every RNA-Puzzles challenge, and (**B**) lasso and interlace-type entanglements found in these models.

In the analyzed dataset of 1,017 non-redundant RNA 3D structures, we have identified 138 instances with entanglements (Figure 3 A), including 137 predicted models and one target—the experimentally determined 3D structure of an exonuclease resistant RNA from Zika virus (PDB ID: 5TPY, PZ18). In the case of seven targets, all submitted RNA 3D models are free of entanglement. For the remaining ones, 4–38% of predictions contain at least one pair of entangled structural elements (Table 1). Most models (68%) contain a single entanglement - the target structure with one lasso belongs to this set. But there are also instances with 2–5 entanglements (Table 2). A total of 104 models include only lassos, 14 models—only interlaces and 20 models—both lassos and interlaces (Supplementary Table S1 in the Supplementary Material).

**Table 1.** Detailed information on the non-redundant test dataset divided by puzzles

				Models	Entanglements	
Puzzle	Target	Length	RNA type	Entangled/total	Number	Types
PZ01	3MEI	46	RNA dimer	0 / 14	0	n/a
PZ02	3P59	100	RNA nanosquare	0 / 12	0	n/a
PZ03	3OWZ	84	Glycine riboswitch	0 / 12	0	n/a
PZ04	3V7E	126	SAM-I riboswitch aptamer	0 / 27	0	n/a
PZ05	4P9R	188	Lariat-capping ribozyme	9 / 24	14	5xL&L, 1xD&L,4xL(D), 4xL(L)
PZ06	4GXY	168	Adenosylcobalamin riboswitch	5 / 34	9	3xD&D, 2xL(D), 1xL(L), 3xD(D)
PZ07	4R4V	370	VS ribozyme	8 / 51	15	1xL&L, 5xD&L, 1xD&D, 4xL(D), 2xL(L), 2xD(D)
PZ08	4L81	96	SAM-I/IV riboswitch	5 / 42	6	1xD&L, 3xL(S), 1xL(D), 1xL(L)
PZ09	5KPY	71	5-hydroxytryptophan aptamer	0 / 32	0	n/a
PZ10	4LCK	171	T-box - tRNA complex	0 / 26	0	n/a
PZ11	5LYS	57	7SK 5′-hairpin riboregulator	0 / 53	0	n/a
PZ12	4QLM	125	Ydao riboswitch	3 / 51	4	3xL(D), 1xL(L)
PZ13	4XW7	71	ZTP riboswitch	9 / 55	13	3xL&L, 1xL(S), 4xL(D), 5xL(L)
PZ14a	5DDO	61	L-glutamine riboswitch (free)	3 / 46	6	4xL(S), 2xL(D)
PZ14b	5DDP	61	L-glutamine riboswitch (bound)	2 / 56	3	2xL(D), 1xL(L)
PZ15	5DI4	68	Hammerhead ribozyme	17 / 70	30	10xL&L, 5xL(S), 14xL(D), 1xL(L)
PZ17	5K7C	62	Pistol ribozyme	35 / 105	50	1xL&L, 32xL(S), 9xL(D), 8xL(L)
PZ18	5TPY	71	Exonuclease resistant RNA	9 / 52	10	9xL(S), 1xL(D)
PZ19	5T5A	62	Twister sister ribozyme	2 / 54	3	3xL(S)
PZ20	5Y85	68	Twister sister ribozyme	2 / 40	2	1xL(S), 1xL(D)
PZ21	5NWQ	41	Guanidine-III riboswitch	9 / 51	9	9xL(S)
PZ24	6OL3	112	Viral non-coding RNA	19 / 88	27	2xL&L, 3xD&L, 10xL(S), 9xL(D), 3xL(L)

**Table 2.** The number of RNA models without and with entanglements

Entanglements per model	0	1	2	3	4	5
No. of models	859	94	32	6	5	1

The total number of entanglements in all the models equals 201 (Table 3). This collection includes 165 lassos, and 36 interlaces (Figure 3 B). Loops are involved in 192 identified issues (32 interlaces and 160 lassos), dinucleotide steps in 75 (14 interlaces and 61 lassos), and single-stranded fragments contribute to 77 entanglements (all of them are lassos). The most common motif is a lasso, in which a loop wraps around the second structural element. Dinucleotide step as the lassoing element occurs rarely and only in the D(D) configuration. Neither D(S) nor D(L) type entanglements have been found in the dataset.

**Table 3.** The number of entanglements found in the analyzed dataset

*Interlaces*			
Class	L&L	D&L	D&D
No. of entanglements	22	10	4
*Lassos*			
Class	L(S)	L(D)	L(L)
Entanglements	77	56	27
Class	D(S)	D(D)	D(L)
No. of entanglements	0	5	0

Loop lassoing a single-stranded fragment, that is, entanglement from the L(S) class, occurs most frequently. Such entanglement has been found in the experimental structure targeted in Puzzle 18 (Supplementary Figure S1 in the Supplementary Material) - the molecule (PDB ID: 5TPY) belonging to exonuclease-resistant RNAs (38). Recent works report on the other structures in this group, where threading of the strand results in forming a lasso likely to have biological significance (39).

We have examined RNA 3D models for the relationship between entanglement occurrence and the properties of structures to contain them. The tests prove no correlation between the occurrence of entanglements and structure size (cf. Supplementary Figure S2 in the Supplementary Material). The same we have found for steric clashes—Clash Score of entangled RNAs ranges from 0 to 176, while eight structures with entanglements have a zero Clash Score value. Thus, the entanglement does not necessarily result in an unnatural overlap of non-bonding atoms. The RMSD of entangled models is between 3.74 and 34.04 Å—for 93%, it is >10 Å. Such high RMSD values are likely due to the appearance of entanglements, which are not present in the native structures. However, there is no direct evidence that more entangled elements in the RNA structure make the larger RMSD and vice versa (Supplementary Figure S3 in the Supplementary Material). RNA-Puzzles assessment tables sorted by increasing RMSD show that the entangled models are not always at the bottom of the ranking (Supplementary Figure S4 in the Supplementary Material).

**Figure 4.**
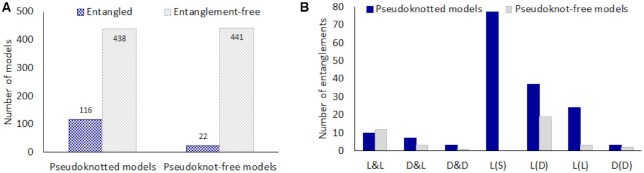
(**A**) The number of entangled and entanglement-free models, and (**B**) the distribution of entanglement types in RNA 3D structures with and without pseudoknots.

Finally, we asked whether pseudoknots affect the occurrence of entanglements. We checked which models in the dataset are pseudoknotted and which ones are entangled, and we determined a product of these subsets. We found pseudoknots in 554 out of 1,017 analyzed non-redundant structures. In this subset, 116 models included entangled structure elements and 438 did not. We also found out 22 entangled models without pseudoknots. The probability *P* of entanglement formation in pseudoknotted structures (*P* = 0.21) appears four times higher than in RNA models without pseudoknots (*P* = 0.05). 84% of entangled models have pseudoknots. We have learned that base pairs forming a pseudoknot often contribute to entanglement. Interestingly, L(S) lassos in our dataset occur only in the pseudoknotted RNA 3D models (Figure 4). The interlace of type D&D is also characteristic of models containing pseudoknots. In general, 73% of lassos and 56% of interlaces from the dataset are formed in the pseudoknotted structures.

### Example predictions with entanglements

Here, we present three examples of RNA 3D models predicted within RNA-Puzzles: model 5 submitted by Chen group in Puzzle 17 (PZ17C5), model 8 submitted by Das group in Puzzle 13 (PZ13D8), and model 1 submitted by Adamiak group in Puzzle 5 (PZ05A1). The examples are representative of the analyzed dataset: each of them comes from a different round of RNA-Puzzles (PZ17C5—Round IV, PZ13D8—Round III, PZ05A1 - Round II), was modeled using the other prediction method (Vfold3D (40), Rosetta (41) and RNAComposer (33), respectively), and contains entanglements of different types.

**Figure 5.**
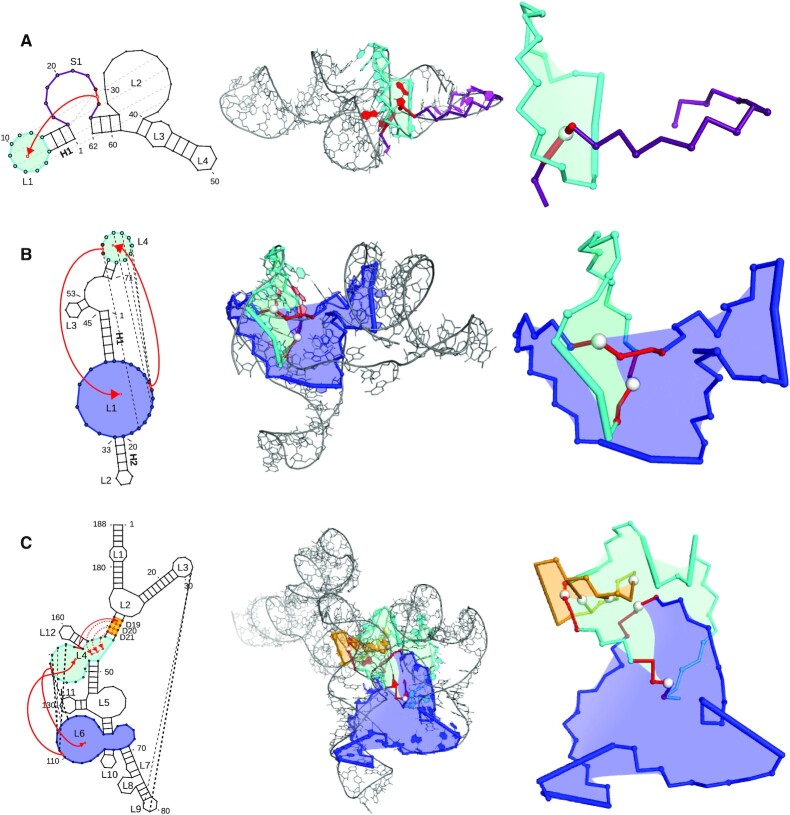
Example RNA 3D structure models predicted within RNA-Puzzles, with entanglements depicted in the secondary and tertiary structure visualization: (**A**) PZ17C5 (62nt) with L(S) entanglement, (**B**) PZ13D8 (71nt) with L&L entanglement, and (**C**) PZ05A1 (188nt) with L&L, L&D and L(D) entanglements.

The first example comes from the puzzle that targeted the 3D structure of the pistol ribozyme (PDB ID: 5K7C) (42). The native 62-nucleotide RNA is not entangled but contains a pseudoknot - its computational modeling may result in the creation of entanglements. Out of 105 non-redundant predictions submitted in this puzzle, 35 contain entangled structure elements. The example PZ17C5 model, with RMSD 9.44 Å, is placed at the seventh position of the RMSD ranking (12). At the same time, stereochemistry analysis using RCSB MAXIT software shows that it has the largest number of close-contact errors among all the submissions to this puzzle. PZ17C5 includes one entanglement classified as L(S). In the secondary structure of this model (Figure 5, panel A), we can see two hairpins connected by a single-stranded, 8-nucleotide long linker (S1). One hairpin is composed of four base pairs forming the double helix (H1) and an 8-nucleotide apical loop (L1). The stem of the other hairpin contains a large asymmetrical internal loop (L2). These two elements, L1 and L2, form a pseudoknot. The pseudoknot helix and H1 are coaxial. In the PZ17C5 model, the S1 linker is threaded through the L1 loop. The puncturing of the area inside L1 falls between the A23 and G24 residues of S1. G24 clashes with G10–G11 residues that belong to L1, whereas A23 forms Watson-Crick hydrogen bonds with U6.

The second example is taken from the set of 55 submissions to Puzzle 13 (all of them non-redundant), challenging prediction of the ZTP riboswitch (PDB ID: 4XW7) with the 71-nucleotide sequence (43). Nine of these models contain entangled structure elements. PZ13D8 represents an entanglement from the L&L category. A look at the secondary structure of this model (Figure 5, panel B) reveals three hairpins. The first one contains a large asymmetrical internal loop (L1) within the stem. It forms a pseudoknot with the apical loop (L4) of the third hairpin. In the 3D structure model, while forming this pseudoknot, the L4 loop encircles the 5′ strand of L1. Consequently, L4 punctures the area within L1 at the C61 site, whereas L1 threads through L4, puncturing its inner area at G13. This entanglement is one of the reasons for the model’s quite large RMSD (13.16 Å), which places it 43rd in the ranking (11). Additionally, PZ13D8 does not show coaxial stacking between H1 and H2 stems, which are adjacent to the internal loop L1, as observable in the crystal structure.

The third example is selected from 24 non-redundant RNA 3D models submitted to Puzzle 5. The lariat-capping ribozyme (PDB ID: 4P9R) with a 188-nucleotide long sequence (44) was a target of this challenge. PZ05A1 belongs to a group of 10 entangled structures. Its RMSD (16.78 Å), although not stunning, is the third-best in the whole collection (10). Let us note that it is by the presence of entanglements that this model has quite a big RMSD value. When compared to the two better models provided by the Das group, it preserves the secondary structure and overall fold. However, Das’s predictions have no entanglements, while PZ05A1 has four of them: L&L, L&D and two L(D) ones. In the secondary structure of PZ05A1 (Figure 5, panel C), we can see four 3-way junctions, L2, L4, L5 and L6. Large single-stranded regions of L4 and L6 form a pseudoknot. In the 3D space, these two loops interlace in the L&L-type conformation. Additionally, the stem between L2 and L4 is punctured between two base pairs (C43–G172, G44–C171) that form dinucleotide step D19 by the L4 loop. Here, we observe entanglement of type L&D. Finally, L4 is lassoing two adjacent dinucleotide steps, D20 and D21, displaying the L(D) conformation.

### Entanglements in experimental structures

Finding an entanglement in the PZ18 target of RNA-Puzzles (PDB ID: 5TPY) motivated us to process with our algorithm some RNA 3D structures from the Protein Data Bank (45). For the analysis, we selected large molecules that have complex structures and potentially many structural elements. The first is a 2904-nucleotides long 23S rRNA structure from *Escherichia coli* (PDB ID: 1C2W), reconstructed by cryo-electron microscopy at 7.5 Å resolution (19). This structure was already studied within the quest for topological knots in RNA molecules (21,22). A knot and four extraordinary clasps were found in it, suspected of being artifacts of the cryo-em reconstruction procedure. Our algorithm identified in it as many as 25 entanglements (Supplementary Table S2 in the Supplementary Material), including two interlaces of type L&L and 23 lassos: 1xL(S), 16xL(D) and 6xL(L). There are 33 different structure elements contributing to the formation of these entanglements—16 loops, 16 dinucleotide steps, and 1 open single-stranded fragment. The L(S) lasso resulting from the loop (C66–G88) entangled with a long single-stranded fragment (A89–G317) forms a topological knot studied in (21,22).

The second example is a 3044-nucleotides long 23S rRNA from *Haloarcula marismortui* (PDB ID: 1FFK). Its 2.4Å resolution structure was determined using X-ray diffraction (46). Although this molecule has a very complex structure, our algorithm identified only one lasso-type L(S) entanglement in it. Two structural elements are involved in the lasso: the apical loop C61–G83 and the long fragment G88-G323. Note that both selected molecules belong to the same RNA type and have similar sequence lengths. Their secondary structures display significant similarities, and in both of them, a pseudoknot motif is present. They differ in resolution and the number of entanglements. The first structure (1C2W) has poor resolution and a large number of entanglements, while the second one - with a good resolution - contains only one entanglement. Thus, it is highly likely that structure elements entanglements are artifacts related to the imprecision of the experimental method and arise in the process of RNA 3D model reconstruction from experimental data. A similar hypothesis concerning topological knot was formulated in (22).

## CONCLUSION

This work presents the problem of entangled elements occurring in RNA 3D structures. The entanglement is defined as the spatial arrangement of two structural elements, where at least one of them punctures the other. Such an arrangement can have an interlaced or lasso topology. An analysis of 1,017 non-redundant RNA 3D structure models from the RNA-Puzzles collection has shown that 14% of the predicted models and one experimentally determined RNA 3D structure included entanglements. Some instances had more than one issue. In total, we identified 201 entanglements—82% of them in the lasso and 18% in the interlaced topology.

In the predicted RNA 3D models, entanglements - the artifacts of computational procedures—come from the overlap of structural elements when assembling the structure from fragments or when transforming a coarse-grained to a full-atomic model. In the experimental structures, they might occur as a result of the model reconstruction from imprecise experimental data. The lack of suitable algorithms to identify entanglements has so far prevented the detection of such conformations and the sifting of entangled RNA 3D structures from the resulting set of models. Therefore, we provide the program developed for this study to enable easy identification and classification of entangled structure elements in RNA 3D models. We suggest the potential application of the program to validate both computationally generated and experimental structures. We hope it will contribute to improving the quality of RNA 3D structure prediction systems. The program, a component of the RNApolis group (47), is available at: https://www.cs.put.poznan.pl/mantczak/spider.zip.

## DATA AVAILABILITY

Computational tests described in the manuscript were performed on a benchmark set with RNA 3D structure models predicted within RNA-Puzzles, available at https://github.com/RNA-Puzzles/standardized_dataset.

## Supplementary Material

gkab716_Supplemental_File
