# Supplementary material for: Entanglements of structure elements revealed in RNA 3D models
Source: Nucleic Acids Res. 2021 Aug 25;49(17):9625–32. doi: 10.1093/nar/gkab716 (PMC8464073; doi:10.1093/nar/gkab716)

Supplementary Material

Entanglements of structure elements revealed in RNA 3D models

M. Popenda[1], T. Zok[2], J. Sarzynska[1], A. Korpeta[2],

R.W. Adamiak[1,2], M. Antczak[1,2,*], M. Szachniuk[1,2,*]

[1] Institute of Bioorganic Chemistry, Polish Academy of Sciences, 61-704 Poznan, Poland

[2] Institute of Computing Science and European Centre for Bioinformatics and Genomics, Poznan University of Technology, 60-965 Poznan, Poland

*To whom correspondence should be addressed:

mantczak@cs.put.poznan.pl, mszachniuk@cs.put.poznan.pl

Table S1. Non-redundant entangled RNA structure 3D models in RNA-Puzzles.

No.	3D RNA model	RMSD [Å]	Clash score	Pseudo-knot	Types of entanglements	Number of punctures
1	PZ05_Adamiak_1	16.78	15.9	+	D&L, L&L, L(D), L(D)	6
2	PZ05_Bujnicki_4	22.24	1.32	+	L&L	2
3	PZ05_Chen_1	27.24	5.46	–	L&L	4
4	PZ05_Chen_3	28.71	11.59	+	L(L)	2
5	PZ05_Chen_4	31.52	8.94	+	L(L), L(L)	4
6	PZ05_Dokholyan_2	19.9	11.42	+	L(L)	2
7	PZ05_Dokholyan_6	20.82	14.4	+	L&L, L(D)	3
8	PZ05_Dokholyan_7	21.44	11.09	+	L&L	2
9	PZ05_Dokholyan_8	24.01	12.09	+	L(D)	2
10	PZ06_Bujnicki_2	30.97	1.28	–	L(D)	2
11	PZ06_Das_1	14.48	28.07	+	D&D, D(D)	3
12	PZ06_Das_4	11.7	27.7	+	D&D, D(D)	3
13	PZ06_Das_6	12.41	28.99	+	D&D, D(D)	3
14	PZ06_Dokholyan_5	22.77	10.64	–	L(D), L(L)	3
15	PZ07_Chen_2	34.04	59.79	–	D&L	2
16	PZ07_Chen_4	27.57	65.65	–	D&D, D&L, L(D)	5
17	PZ07_Chen_9	32.74	64.15	–	D(D), D(D), D&L, L(D)	6
18	PZ07_Das_4	25	9.58	+	L(D)	2
19	PZ07_Major_2	27.91	51.94	+	D&L, L(D)	4
20	PZ07_Major_3	26.66	56.3	+	D&L	2
21	PZ07_Major_8	27.15	51.52	+	L(L)	2
22	PZ07_Major_9	27.12	67.13	+	L&L, L(L)	4
23	PZ08_Bujnicki_8	11.48	5.76	+	L(S)	1
24	PZ08_Bujnicki_10	10.8	17.61	+	L(S)	1
25	PZ08_Chen_1	13.08	11.85	+	D&L	2
26	PZ08_Das_5	10.81	7.7	+	L(S)	1
27	PZ08_Dokholyan_3	24.75	6.4	–	L(D), L(L)	3
28	PZ12_Ding_4	14.05	13.11	+	L(D)	2

No.	3D RNA model	RMSD [Å]	Clash score	Pseudo-knot	Types of entanglements	Number of punctures
29	PZ12_Ding_6	15.41	15.33	+	L(D)	2
30	PZ12_Xiao_2	29.56	50.47	–	L(D), L(L)	4
31	PZ13_Bujnicki_1	8.97	37.38	+	L(D), L(D), L(L)	4
32	PZ13_Bujnicki_3	8.38	22.13	+	L(L)	2
33	PZ13_Bujnicki_4	14.56	41.25	+	L(D), L(D), L(L)	4
34	PZ13_Bujnicki_9	16.99	1.74	+	L(S)	1
35	PZ13_Bujnicki_10	14.84	3.04	+	L&L	2
36	PZ13_Das_3	8.87	6.07	+	L(L)	2
37	PZ13_Das_4	11.01	8.68	+	L(L)	2
38	PZ13_Das_8	13.16	4.77	+	L&L	2
39	PZ13_Xiao_7	27.59	32.16	–	L&L	2
40	PZ14_BujnickiPostExp_1	13.24	14.74	+	L(S), L(S)	2
41	PZ14_BujnickiPostExp_3	15.98	12.7	+	L(D), L(S), L(S)	5
42	PZ14_BujnickiPostExp_4	12.69	28.4	+	L(D)	2
43	PZ14_BujnickiPreExp_7	15.28	8.61	+	L(D), L(L)	4
44	PZ14_DingPreExp_1	9.51	11.68	+	L(D)	2
45	PZ15_3dRNAAS2_1	24.68	35.08	–	L&L, L(D)	4
46	PZ15_3dRNAAS2_2	24.57	33.71	–	L&L, L(D)	4
47	PZ15_3dRNAAS2_3	24.6	32.8	–	L&L, L(D)	4
48	PZ15_3dRNAAS2_4	24.58	32.8	–	L&L, L(D)	4
49	PZ15_3dRNAAS2_5	24.59	32.35	–	L&L, L(D)	4
50	PZ15_3dRNAAS2_6	24.63	31.89	–	L&L, L(D)	4
51	PZ15_3dRNAAS2_7	24.59	32.8	–	L&L, L(D)	4
52	PZ15_3dRNAAS2_8	24.78	34.62	–	L&L, L(D)	4
53	PZ15_3dRNAAS2_9	24.6	34.62	–	L&L, L(D)	4
54	PZ15_3dRNAAS2_10	24.64	34.17	–	L&L, L(D)	4
55	PZ15_Chen_8	12.1	1.36	+	L(S)	1
56	PZ15_Chen_10	9.35	0.91	+	L(S)	1
57	PZ15_SimRNAAS1_1	7.12	94.72	+	L(S)	1
58	PZ15_SimRNAAS1_2	20.67	96	+	L(D), L(D)	3
59	PZ15_SimRNAAS1_4	24.15	88.76	+	L(S)	1

No.	3D RNA model	RMSD [Å]	Clash score	Pseudo-knot	Types of entanglements	Number of punctures
60	PZ15_SimRNAAS1_5	20.89	85.23	+	L(S)	1
61	PZ15_SimRNAAS1_9	19.08	64.78	+	L(D), L(D), L(L)	4
62	PZ17_Bujnicki_1	13.98	3.52	+	L(L)	2
63	PZ17_Bujnicki_2	14.38	0	+	L(L)	2
64	PZ17_Bujnicki_4	16.08	0	+	L(L)	4
65	PZ17_Bujnicki_5	13.95	5.03	+	L(S)	1
66	PZ17_Bujnicki_6	14.35	0	+	L(S)	1
67	PZ17_Bujnicki_7	10.86	1.01	+	L(S)	1
68	PZ17_Bujnicki_8	12.23	0	+	L(S)	1
69	PZ17_Chen_1	16.11	101.96	+	L(L), L(S)	3
70	PZ17_Chen_3	17.35	0	+	L(D), L(L), L(S), L(S)	5
71	PZ17_Chen_5	9.44	157.15	+	L(S)	1
72	PZ17_Chen_6	14.75	176.17	+	L(S)	1
73	PZ17_Chen_7	16.45	4.52	+	L(L), L(S)	4
74	PZ17_Chen_8	16.61	1.51	+	L(L), L(D), L(S)	5
75	PZ17_Chen_9	15.6	107.14	+	L(D), L(L)	4
76	PZ17_Das_5	15.38	8.04	+	L(S)	1
77	PZ17_Das_6	11.51	4.02	+	L(S)	1
78	PZ17_Das_10	17.44	14.07	+	L&L, L(S), L(S), L(S)	5
79	PZ17_DasExtraInfo_1	11.02	10.05	+	L(S)	1
80	PZ17_DasExtraInfo_2	12.07	10.05	+	L(S)	1
81	PZ17_DasExtraInfo_3	13.23	13.07	+	L(S)	1
82	PZ17_Ding_1	15.02	8.04	+	L(D)	2
83	PZ17_Ding_2	16.04	11.06	+	L(D), L(S)	3
84	PZ17_Ding_3	14.51	10.05	+	L(D), L(S)	3
85	PZ17_Ding_4	15.75	10.05	+	L(D)	2
86	PZ17_Ding_5	16.02	8.54	+	L(D)	2
87	PZ17_Ding_6	16.93	10.05	+	L(S)	1
88	PZ17_Ding_7	12.86	9.05	+	L(S)	1
89	PZ17_Ding_10	13.39	8.54	+	L(S)	1

No.	3D RNA model	RMSD [Å]	Clash score	Pseudo-knot	Types of entanglements	Number of punctures
90	PZ17_Major_2	18.04	0	–	L(D)	2
91	PZ17_RNAComposerAS2_5	12.56	11.07	+	L(S)	1
92	PZ17_SimRNAAS1_5	17.01	157.92	+	L(S)	2
93	PZ17_SimRNAAS2_3	15.59	124.87	+	L(S), L(S)	3
94	PZ17_SimRNAAS2_5	18.59	130.02	+	L(S), L(S)	4
95	PZ17_SimRNAAS2_6	12.2	119.78	+	L(S)	1
96	PZ17_SimRNAAS2_8	12.77	152.96	+	L(S)	1
97	PZ18_target	0	3.48	+	L(S)	1
98	PZ18_Chen_1	3.74	0.43	+	L(S)	1
99	PZ18_Chen_2	6.64	2.17	+	L(S)	1
100	PZ18_Das_1	5.5	13.05	+	L(S)	1
101	PZ18_Das_2	3.88	16.09	+	L(S)	1
102	PZ18_Das_3	5.63	9.57	+	L(S)	1
103	PZ18_Das_4	5.7	13.05	+	L(S)	1
104	PZ18_Dokholyan_1	8.44	9.57	+	L(S_3)	3
105	PZ18_Dokholyan_2	11.95	14.79	+	L(S)	1
106	PZ18_RNAComposer_5	15.21	13.92	–	L(D)	2
107	PZ19_Das_1	15.26	14.48	+	L(S)	1
108	PZ19_Das_4	10.78	12.98	+	L(S), L(S)	4
109	PZ20_RNAComposer_3	12.64	10.03	+	L(D)	2
110	PZ20_simRNA_3	22.12	97.22	–	L(S)	1
111	PZ21_Bujnicki_2	8.67	24.79	+	L(S)	1
112	PZ21_Bujnicki_3	11.94	0	+	L(S)	1
113	PZ21_DasLORES_3	14.24	6.02	+	L(S)	1
114	PZ21_RNAComposer_3	12.12	18.05	+	L(S)	1
115	PZ21_RNAComposer_4	12.99	9.02	+	L(S)	1
116	PZ21_simRNA_1	11.85	116.45	+	L(S)	1
117	PZ21_simRNA_2	11.48	116.54	+	L(S)	1

No.	3D RNA model	RMSD [Å]	Clash score	Pseudo-knot	Types of entanglements	Number of punctures
118	PZ21_simRNA_3	13.11	117.56	+	L(S)	1
119	PZ21_simRNA_4	10.57	118.53	+	L(S)	1
120	PZ24_DasTFN_2	13.3	4.99	+	L(S)	1
121	PZ24_DasTFN_3	16.77	1.66	+	L(S)	1
122	PZ24_DasTFN_4	21.89	1.94	+	L(S)	1
123	PZ24_DasTFN_5	19.59	4.16	+	L(S)	1
124	PZ24_FARFAR2_2	16.87	0.55	+	L(S)	1
125	PZ24_FARFAR2_3	19.08	0.83	+	L(S)	1
126	PZ24_FARFAR2_5	18.41	0.83	+	L(S)	2
127	PZ24_Kollmann_1	30.15	115.47	+	L&L	4
128	PZ24_Kollmann_3	32.86	120.66	+	L&L	2
129	PZ24_Kollmann_6	30.52	92.12	+	L(D), L(L)	3
130	PZ24_Kollmann_8	32.43	114.07	+	L(S)	2
131	PZ24_SimRNA_3	13.74	124.86	+	L(D), L(D), L(D), L(D), L(L)	6
132	PZ24_SimRNA_5	15.38	96	+	L(D), L(D), L(D), L(L)	6
133	PZ24_Vfold3D_3	15.5	1.94	+	L(S)	1
134	PZ24_VfoldLA_1	15	144.71	+	D&L	2
135	PZ24_VfoldLA_2	15.35	171.09	+	D&L	2
136	PZ24_VfoldLA_3	14.32	158.98	+	L(S)	1
137	PZ24_VfoldLA_5	13.65	147.87	+	D&L	2
138	PZ24_iFoldRNA_4	27.07	1.94	+	L(D)	2

Table S2. Entanglements identified in 23S rRNA structure from *E. coli* (PDB ID: 1C2W).

No.	Class	Topology	Element 1	Element 2
1	L&L	Interlace	C274-C281, G359-G363	C239-G245, C253-G258
2	L(L)	Lasso	G297-G301, C316-G325, C337-G341	G327-C335
3	L(L)	Lasso	C584-U589,A668-C671, G809-C812, G1195-U1198, A1247-G1256	C672-C678, G799-G808
4	L(L)	Lasso	C1270-A1276, U1294-C1295, G1645-U1648, A2009-G2010	G1281-C1289
5	L(L)	Lasso	A1307-C1314, G1338-C1345, G1601-C1611, G1620-U1621	C1298-G1303, C1625-G1627, C1639-G1642
6	L(L)	Lasso	A1347-C1349, G1382-A1385, U1402-U1405, A1597-U1599	A1307-C1314, G1338-C1345, G1601-C1611, G1620-U1621
7	L&L	Interlace	G1450-C1461	G1444-C1446, G1465-C1472, G1519-G1530, C1541-C1547
8	L(L)	Lasso	C1414-A1420, U1578-G1588	A1630-U1636
9	L(D)	Lasso	C274-C281, G359-G363	U235-C236, G261-A262
10	L(D)	Lasso	C274-C281, G359-G363	C236-C237, G260-G261
11	L(D)	Lasso	C274-C281, G359-G363	C237-C238, G259-G260
12	L(D)	Lasso	C274-C281, G359-G363	C238-C239, G258-G259
13	L(D)	Lasso	C584-U589, A668-C671, G809-C812, G1195-U1198, A1247-G1256	C16-G17, C523-G524
14	L(D)	Lasso	C584-U589, A668-C671, G809-C812, G1195-U1198, A1247-G1256	A590-U591, A666-U667
15	L(D)	Lasso	C1270-A1276, U1294-C1295, G1645-U1648, A2009-G2010	G1280-G1281, C1289-C1290
16	L(D)	Lasso	A1307-C1314, G1338-C1345, G1601-C1611, G1620-U1621	G1296-C1297, G1643-C1644
17	L(D)	Lasso	A1307-C1314, G1338-C1345, G1601-C1611, G1620-U1621	C1297-C1298, G1642-G1643
18	L(D)	Lasso	A1307-C1314, G1338-C1345, G1601-C1611, G1620-U1621	G1303-A1304, U1624-C1625
19	L(D)	Lasso	A1307-C1314, G1338-C1345, G1601-C1611, G1620-U1621	A1304-C1305, G1623-U1624
20	L(D)	Lasso	A1307-C1314, G1338-C1345, G1601-C1611, G1620-U1621	C1305-C1306, G1622-G1623

No.	Class	Topology	Element 1	Element 2
21	L(D)	Lasso	A1347-C1349, G1382-A1385, U1402-U1405, A1597-U1599	C1345-G1346, C1600-G1601
22	L(D)	Lasso	C1414-A1420, U1578-G1588	U1629-A1630, U1636-A1637
23	L(D)	Lasso	G1444-C1446, G1465-C1472, G1519-G1530, C1541-C1547	C1447-G1448, C1463-G1464
24	L(D)	Lasso	G1444-C1446, G1465-C1472, G1519-G1530, C1541-C1547	G1448-G1449, C1462-C1463
25	L(S)	Lasso	C66-G88	A89-G317

Figure S1. Experimental structure targeted in Puzzle 18 (PDB ID: 5TPY) with L(S)-type entanglement. The dangling 5'-end is threaded through the loop surface represented by a yellow shaded area. The grey bead in the 3D visualization marks the puncture site.

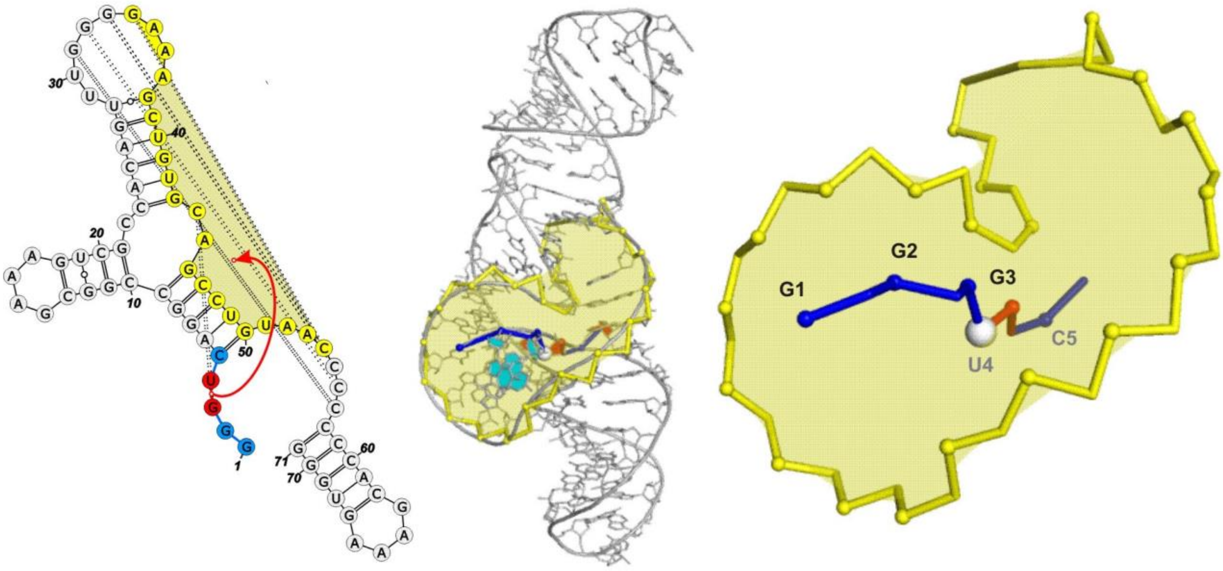

Figure S2. The length of the analyzed RNA 3D structure models vs the number of entangled structure elements found in these models. Spearman correlation coefficient: 0.03.

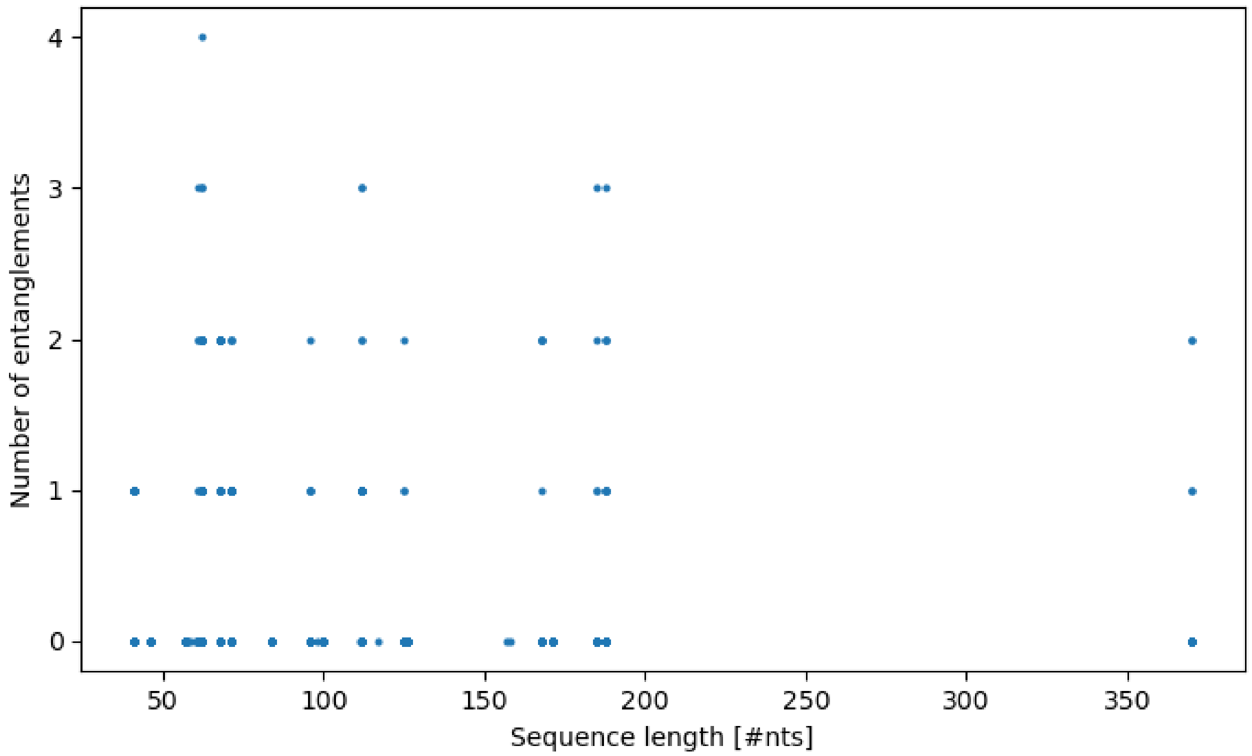

Figure S3. RMSD of the analyzed RNA 3D structure models vs the number of entangled structure elements found in these models. Spearman correlation coefficient: 0.13.

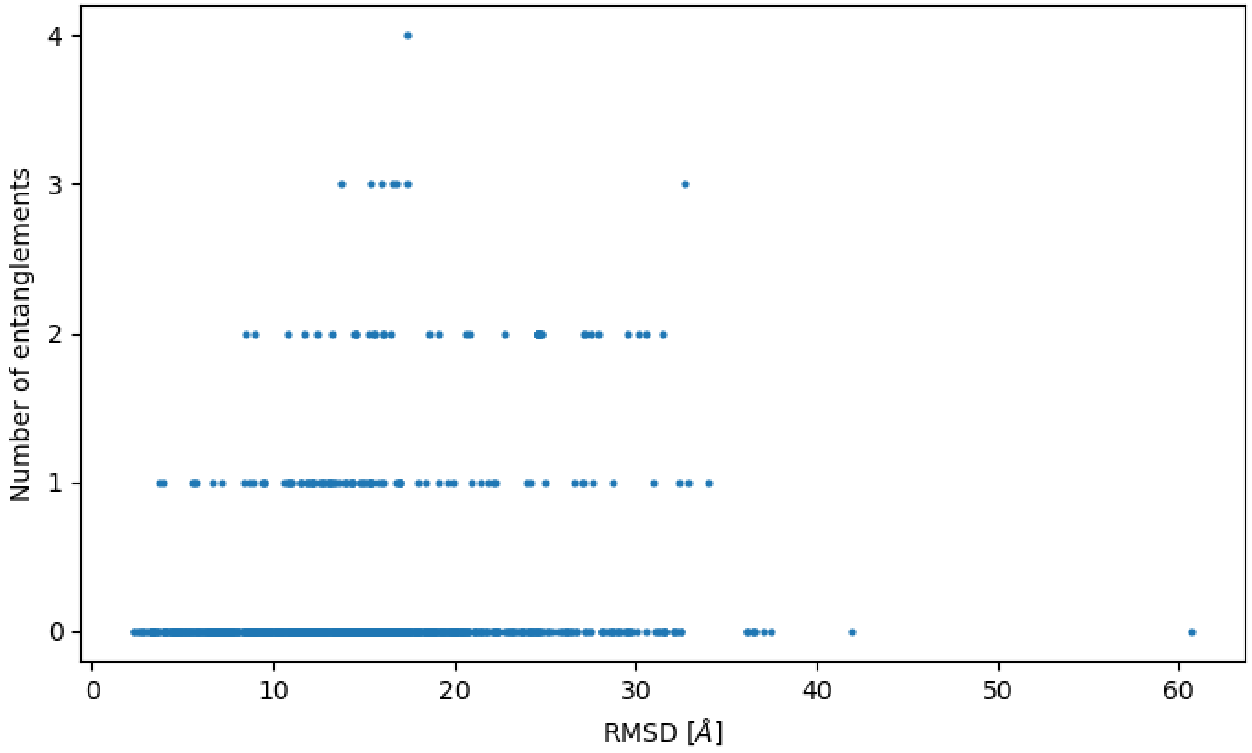

Figure S4. Normalized RMSD-driven rank of the analyzed RNA 3D structure models vs the number of entangled structure elements found in these models. The lower the RMSD rank – the higher is the model positioning in the ranking. Spearman correlation coefficient: 0.005.

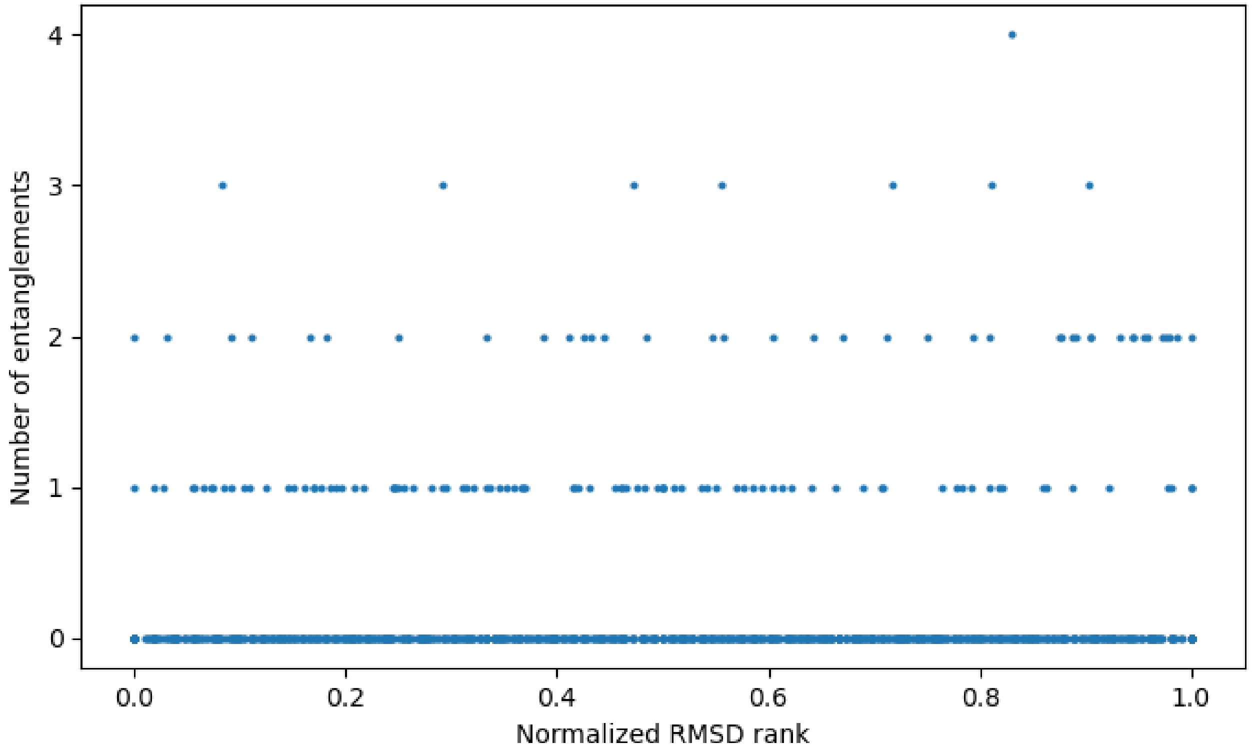

Supplement: gkab716_Supplemental_File [file gkab716_supplemental_file.pdf]
